# Neuroplasticity in addiction: cellular and transcriptional perspectives

**DOI:** 10.3389/fnmol.2012.00099

**Published:** 2012-11-12

**Authors:** Heather B. Madsen, Robyn M. Brown, Andrew J. Lawrence

**Affiliations:** ^1^Addiction Neuroscience Laboratory, Florey Institute of Neuroscience and Mental HealthParkville, VIC, Australia; ^2^Centre for Neuroscience Research, University of MelbourneParkville, VIC, Australia

**Keywords:** addiction, plasticity, CREB, deltaFosB, epigenetics, histone modification, DNA methylation, microRNAs

## Abstract

Drug addiction is a chronic, relapsing brain disorder which consists of compulsive patterns of drug-seeking and taking that occurs at the expense of other activities. The transition from casual to compulsive drug use and the enduring propensity to relapse is thought to be underpinned by long-lasting neuroadaptations in specific brain circuitry, analogous to those that underlie long-term memory formation. Research spanning the last two decades has made great progress in identifying cellular and molecular mechanisms that contribute to drug-induced changes in plasticity and behavior. Alterations in synaptic transmission within the mesocorticolimbic and corticostriatal pathways, and changes in the transcriptional potential of cells by epigenetic mechanisms are two important means by which drugs of abuse can induce lasting changes in behavior. In this review we provide a summary of more recent research that has furthered our understanding of drug-induced neuroplastic changes both at the level of the synapse, and on a transcriptional level, and how these changes may relate to the human disease of addiction.

## Introduction

Drug addiction is a chronic, relapsing disorder characterized by uncontrolled, compulsive drug use that persists despite serious negative consequences. One of the most insidious features of addiction is the enduring susceptibility to relapse displayed by users despite months or even years of abstinence (O'Brien, 1997). Importantly, not everyone who uses drugs becomes addicted, and whether or not a person makes this transition can be influenced by a complex interplay of genetic and environmental factors (Goldman et al., 2005; Kendler et al., 2007). The escalation of drug use from casual to compulsive and the persistent vulnerability to relapse is thought to be underpinned by long-lasting neuroadaptations in brain reward circuits (Thomas et al., 2008; Luscher and Malenka, 2011; Robison and Nestler, 2011). Essentially all drugs of abuse exert their acute reinforcing properties via the mesocorticolimbic dopamine pathway, encompassing dopamine neurons that originate in the ventral tegmental area (VTA) and project to the striatum and other limbic regions including the prefrontal cortex (PFC), amygdala and hippocampus (Di Chiara and Imperato, 1988; Le Moal and Simon, 1991). The striatum also receives glutamatergic input from the PFC, and while mesolimbic dopamine is no doubt important for the initial stages of drug-taking and reinforcement, a role for corticostriatal glutamate transmission in the compulsive and enduring nature of addiction is being increasingly recognized (Kalivas, 2009; Kalivas et al., 2009). A major focus of research at present lies in characterizing the cellular and molecular changes that occur within this motivational circuitry to contribute to the development and persistence of addiction. In the laboratory, various behavioral facets of addiction can be investigated using animal models (summarized in Table 1). The purpose of this review is to provide an overview of the neuroplastic changes that occur both at the synapse, and on the level of gene transcription, that contribute to addiction-related behaviors.

**Table 1:** **Modeling addiction in animals**.

**Locomotor sensitization:** Locomotor sensitization describes the progressive increase in locomotor activity that usually follows repeated, intermittent drug exposure. Sensitization can persist for months or even years following withdrawal, and as such it is considered to be an indication of enduring drug-induced plasticity (Steketee, 2003). Although it is most commonly studied in relation to psychostimulants, sensitization has also been characterized in response to opiates, nicotine and ethanol (Shuster et al., 1977; Kalivas and Duffy, 1987; Robinson et al., 1988; Benwell and Balfour, 1992; Cunningham and Noble, 1992). Cross-sensitization between different drugs of abuse has also been shown to exist, suggesting that common mechanisms underlie the development of this phenomenon despite these drugs having distinct pharmacological actions in the brain (Vezina and Stewart, 1990; Itzhak and Martin, 1999; Beyer et al., 2001; Cadoni et al., 2001).
**Conditioned place preference (CPP):** CPP is an indirect measure of drug reward based on classical (Pavlovian) conditioning principles (Tzschentke, 1998). The CPP apparatus consists of two distinct environments, one of which is paired with a drug, and with repeated pairing the drug-paired environment acquires secondary motivational properties which can elicit approach behavior. An animal is said to have obtained a place preference if it spends more time in the drug-paired environment when given a choice. This paradigm is used to measure conditioned drug reward and associative learning.
**Operant self-administration:** Animals can be trained to self-administer most drugs that are commonly abused by humans. This is usually achieved using operant boxes where an instrumental task such as a lever press or nose poke results in the delivery of a drug or natural reward. Reward delivery can be paired with a discrete cue such as a tone or light, or passive contextual cues.
**Extinction/reinstatement:** Extinction describes a reduction in conditioned drug-seeking behavior after it is repeatedly non-reinforced (Myers and Davis, 2002). Extinction can be performed in the context of CPP, where an animal is repeatedly exposed to the drug-paired environment in the absence of the drug. Once a CPP is extinguished, it can be reinstated by drug priming (Mueller and Stewart, 2000) or exposure to stressors (Sanchez and Sorg, 2001; Wang et al., 2006). Operant self-administration behavior can also be extinguished by removal of drug reinforcement, and subsequently reinstated by non-contingent exposure to the drug (Dewit and Stewart, 1981), exposure to cues or contexts previously associated with the drug (Meil and See, 1996; Weiss et al., 2000; Crombag and Shaham, 2002), or exposure to stress (Shaham and Stewart, 1995; Erb et al., 1996; Shepard et al., 2004). These same factors are known to precipitate drug craving and relapse in human addicts, and as such reinstatement attempts to model relapse-like behavior in animals.

## Synaptic plasticity mechanisms: addiction as a pathological form of learning and memory

The observation that drug-taking and relapse are quite often directly linked to exposure to drug-related cues highlights the importance of associative learning mechanisms in addiction (Wikler and Pescor, 1967; Tiffany and Drobes, 1990; O'Brien et al., 1998). Steven Hyman made the point that “memory disorders are often thought of as conditions involving memory loss, but what if the brain remembers too much or too powerfully records pathological associations?” (Hyman, 2005). In this context, addiction can be perceived, at least in part, as a pathological form of learning and memory. In support of this hypothesis research over the last decade has demonstrated that drugs of abuse do indeed modify synaptic plasticity in the mesocorticolimbic and corticostriatal circuitry by similar mechanisms that underlie long-term memory formation. What these modifications actually represent in terms of behavior and addiction more generally is another, perhaps more challenging, question. The following section will overview the synaptic adaptations caused by drugs of abuse as measured electrophysiologically in the context of animal models and their relevance to the addicted state.

It was Santiago Ramon y Cajal who, over 100 years ago, contemplated the idea that changes in the strength of synaptic connections between neurons could be the way in which the brain stores information (Cajal, 1894). The discovery of long-term potentiation (LTP) in the hippocampus in 1973 provided the first evidence that this may be the case (Bliss and Lomo, 1973). LTP is the enhancement of synaptic strength that results from synchronous firing of connecting neurons, whereas its counterpart long-term depression (LTD) is the weakening of synaptic strength (Citri and Malenka, 2008). These processes usually involve N-methyl-D-aspartate (NMDA) receptor-mediated trafficking of α-amino-3-hydroxyl-5-methyl-4-isoxazole-propionate (AMPA) receptors to and from the cell surface (Kauer and Malenka, 2007). An NMDA receptor-mediated increase in calcium levels in the postsynaptic cell is required for the induction of LTP and LTD, with the amount of calcium determining the sequence of events. Large increases in calcium preferentially activate protein kinases and result in LTP, ultimately expressed as enhanced transmission at postsynaptic AMPA receptors. In contrast, more modest increases in calcium preferentially activate protein phosphatases and produce LTD, which is expressed as a decrease in AMPA receptor transmission (Kauer and Malenka, 2007). While LTP and LTD were initially studied in relation to learning and memory in the hippocampus, they are now known to occur at most excitatory synapses throughout the central nervous system, and are important for many forms of experience-dependent plasticity (Malenka and Bear, 2004; Kauer and Malenka, 2007).

### Drug-evoked potentiation at excitatory synapses in the VTA

A pioneering study by Ungless and colleagues in 2001 demonstrated that a single exposure to cocaine caused an enhancement of synaptic strength at excitatory synapses on VTA DA neurons when measured 24 h later in brain slices (Ungless et al., 2001). This was measured as an increase in the ratio of AMPA-mediated excitatory postsynaptic currents (EPSCs) over NMDA-mediated EPSCs (termed the AMPA/NMDA ratio). Subsequent electrically-evoked LTP was shown to be occluded at excitatory VTA synapses in cocaine-treated mice whereas LTD was enhanced. These observations as well as a number of other electrophysiological measures indicated that the change in plasticity observed potentially shared similar mechanisms to synaptically-evoked LTP (Ungless et al., 2001). It has since been shown that administration of other drugs of abuse including amphetamine, morphine, ethanol, nicotine, and benzodiazepines can also induce increases in synaptic strength in the VTA, an effect that is not seen with psychoactive drugs that do not have abuse potential (Saal et al., 2003; Gao et al., 2010; Tan et al., 2010). This observation demonstrates a convergence of cellular responses within the VTA by all abused drugs and provides a possible neural mechanism by which initial neuroadaptations underlying addiction could be triggered.

The effect of non-contingent drug administration on VTA synaptic plasticity is transiently expressed, lasting at least 5 but less than 10 days and has been shown to positively correlate with the initial development of behavioral sensitization but not with its expression (Ungless et al., 2001; Saal et al., 2003; Borgland et al., 2004). If cocaine is self-administered the outcome is rather different as plasticity in the VTA becomes persistent and can be detected even 90 days into withdrawal (Chen et al., 2008). The potentiation of glutamatergic synapses on VTA DA cells is presumably linked to the ability of drugs of abuse to enhance extracellular DA in the NAc (Di Chiara and Imperato, 1988) and potentially represents the initiation of “pathological” reward learning whereby a “stamping in” of drug-cue associations occurs. Indeed, NMDA receptor-dependent increases in glutamatergic synaptic strength have been reported in VTA DA neurons during the acquisition of a cue-reward association (Stuber et al., 2008) and recently it was confirmed that cocaine selectively increases the AMPA/NMDA ratio of VTA neurons which project to the NAc as opposed to the PFC (Lammel et al., 2011); it is well established that dopamine transmission within the NAc is critical for the acquisition of a Pavlovian association (Kelley, 2004). Thus it may be that that potentiation of VTA DA neurons may represent neural coding similar to LTP, possibly an associative learning process, which may be essential for early cocaine-induced behavioral responses and has the capacity to trigger long-term adaptations that underlie addiction, though does not represent the addicted state itself. As proposed by others, it may be that addictive drugs co-opt brain reward circuitry to “overlearn” the value of a drug to the organism (Kauer and Malenka, 2007).

The origins of the pertinent glutamatergic projections to the VTA involved in drug-induced plasticity remain to be fully elucidated. One study has revealed that VTA glutamatergic synapses targeted by projections from both the VTA itself and the pedunculopontine nucleus (PPN) show enhanced potentiation from cocaine yet only the synapses receiving input from PPN afferents are potentiated with Δ^9^-tetrahydrocannabinol (THC) (Good and Lupica, 2010). Thus, it appears that the particular glutamatergic afferents involved in drug-induced potentiation can vary according to the drug in question and it may also be the case that a particular projection is common to all drug-evoked excitatory plasticity in the VTA; the latter is yet to be determined. The VTA receives extensive projections from multiple brain regions including the PFC, amygdala and subthalamic nucleus (Geisler and Wise, 2008), many of which have been shown to influence the burst firing of VTA DA neurons (Grillner and Mercuri, 2002). Future experiments utilizing optogenetic techniques could assist in determining the particular projections responsible the drug-evoked potentiation at VTA synapses observed in response to various drugs of abuse, thus shedding light on the on the exact nature of this neuroadaptation.

### Mechanisms underlying drug-evoked synaptic plasticity at excitatory synapse in the VTA

As with electrically-induced LTP in midbrain DA neurons the increase in synaptic strength in the VTA induced by both cocaine and nicotine has been shown to be dependent on NMDA receptor activation (Bonci and Malenka, 1999; Ungless et al., 2001; Mao et al., 2011). In contrast, the maintenance of cocaine-evoked potentiation was recently shown to require the activity of protein kinase Mζ (Ho et al., 2012), an autonomously active protein kinase C (PKC) isoform, whereas spike timing-dependent LTP in VTA DA neurons of drug-naïve mice depends on conventional PKC isoforms (Luu and Malenka, 2008). In the case of nicotine the VTA synaptic potentiation requires the excitation of DA neurons mediated by somatodendritic α4β2 nicotinic acetylcholine receptors (nAChRs) (Mao et al., 2011). Nicotine-induced increases of presynaptic glutamate release also contribute to the induction of this particular synaptic plasticity, likely through increased activation of NMDA receptors (Mao et al., 2011).

Relatively more is known about the mechanisms underlying cocaine-evoked synaptic plasticity than that underlying plasticity induced by other drugs of abuse. Cocaine application to midbrain slices results in potentiation of NMDA receptor transmission within minutes and is proposed to be via insertion of NR2B-containing NMDARs into synapses through a mechanism that requires activation of D_5_ receptors and new protein synthesis (Schilstrom et al., 2006; Argilli et al., 2008). Orexin A has also been shown to be required for both rapid cocaine-induced insertion of NR2B-containing receptors and increased AMPA/NMDA ratios; accordingly the orexin_1_ receptor antagonist SB334867 has been shown to prevent the development of sensitization to cocaine (Borgland et al., 2006). In addition to changes in NMDA receptor subunit expression, increased levels of GluR1-containing (GluR2-lacking) AMPA receptors at synapses have been observed as soon as 3 h after cocaine exposure (Argilli et al., 2008). This observation combined with other recent evidence has led to the hypothesis that synaptic insertion of high-conducting GluR2-lacking receptors contribute to expression of cocaine-induced synaptic potentiation in the VTA (Dong et al., 2004; Bellone and Luscher, 2006; Mameli et al., 2007; Brown et al., 2010; Mameli et al., 2011), for reviews see (Kauer and Malenka, 2007; Wolf and Tseng, 2012). This insertion of GluR2-lacking AMPA receptors depends on NMDA receptor transmission in VTA DA neurons since it is absent in mice lacking functional NMDA receptors in DA neurons (Engblom et al., 2008; Mameli et al., 2009). Insertion of GluR2-lacking AMPA receptors is significant because they have unique properties; they are calcium permeable, have greater single channel conductance than GluR2-containing receptors, and therefore have a huge capacity to alter synaptic transmission (Isaac et al., 2007). Hence, insertion of GluR2-lacking AMPA receptors in the VTA represents a possible mechanism by which drugs of abuse can instantiate the plastic adaptations underlying the initial stages of drug use.

The insertion of GluR2-lacking AMPA receptors into VTA excitatory synapses has now been shown to occur in response to administration of drugs from multiple classes such as nicotine and morphine as well as upon optogenetic activation of DA VTA neurons (Brown et al., 2010). This has led to the proposal that insertion of calcium-permeable GluR2-lacking AMPA receptors represents a universal mechanism which may underlie drug-evoked potentiation of VTA synapses (Brown et al., 2010), though the data for amphetamine are not necessarily consistent with this hypothesis (Faleiro et al., 2004). Moreover, as GluR2-lacking AMPA receptors are inwardly rectifying and thus conduct very little current at +40 mV, their insertion alone cannot explain drug-evoked increases in AMPA/NMDA ratios. A recent study which measured unitary synaptic responses evoked by a highly localized glutamate source (two-photon photolysis of caged glutamate) showed, than in addition to affecting AMPA receptor-mediated EPSCs, cocaine exposure also decreased unitary NMDA receptor-mediated EPSCs (Mameli et al., 2011), thus providing a possible mechanism by which AMPA/NMDA ratios could be increased in this scenario (by lowering the denominator of the ratio). This is yet to be investigated with other drugs of abuse.

The drug-induced exchange of GluR2-containing with GluR2-lacking AMPA receptors can be reversed by activation of mGluR1 receptors in the VTA (Bellone and Luscher, 2006; Mameli et al., 2007). Thus, mGluR1-mediated exchange of AMPA receptors provides a mechanism which can explain why drug-evoked potentiation of VTA synapses is transient in nature, lasting 5 but not 10 days (Ungless et al., 2001; Mameli et al., 2007). Indeed, if mGluR1 function in the VTA is reduced 24 h before cocaine administration then cocaine-induced inward rectification persists beyond 7 days (Mameli et al., 2007, 2009). Hence one possible explanation for why cocaine-evoked synaptic strengthening persists in the VTA following self-administration of cocaine (unlike following non-contingent administration) could be that cocaine self-administration leads to depression of mGluR1 signaling in the VTA.

### Drug–evoked synaptic plasticity at inhibitory synapses in the VTA

Excitatory synapses are not the only type of synapse in VTA DA neurons which are affected by non-contingent administration of drugs of abuse. Inhibitory synapses in the VTA also have a critical role in controlling the firing rate of DA neurons, thus plasticity at GABAergic synapses has the capacity to dramatically influence DA transmission. Indeed, cocaine, morphine and ethanol can all influence inhibitory synaptic plasticity in the VTA (Melis et al., 2002; Liu et al., 2005; Nugent et al., 2007). Repeated cocaine exposure *in vivo* for 5–7 days causes a reduction in amplitudes of GABA-mediated synaptic currents, thereby facilitating LTP induction in VTA cells by reducing the strength of GABAergic inhibition (Liu et al., 2005). Subsequent studies reveal the mechanism of this inhibition to be endocannabinoid-dependent LTD at GABAergic synapses involving activation of ERK1/2 (Pan et al., 2008, 2011). GABA_A_ receptor synapses on VTA dopamine neurons also exhibit robust NMDA-dependent LTP (termed LTP_GABA_) in response to high-frequency stimulation (Nugent et al., 2007). This LTP_GABA_ is absent in VTA slices 2 and/or 24 h after *in vivo* administration of morphine, nicotine, cocaine or ethanol (Nugent et al., 2007; Guan and Ye, 2010; Niehaus et al., 2010). In the case of ethanol the prevention of LTP_GABA_ is mediated by the μ-opioid receptor (Guan and Ye, 2010) Together with synaptic potentiation at excitatory synapses, this loss of LTP_GABA_ should increase the firing of VTA DA neurons following drug exposure.

Slow GABA transmission has also recently been shown to be affected by drugs of abuse. Thus a single dose of methamphetamine or cocaine is sufficient to significantly weaken the ability of GABA_B_ receptors to control VTA GABA neuron firing when measured *ex vivo* 24 h later (Padgett et al., 2012). The methamphetamine-induced loss of the slow inhibitory postsynaptic potential (IPSC) arises from a reduction in GABA_B_ receptor-G protein-coupled inwardly-rectifying potassium channel (GIRK) currents, due to changes in protein trafficking, and is accompanied by a significant decrease in the sensitivity of presynaptic GABA_B_ receptors in GABA neurons of the VTA. Unlike drug-induced influences on GABA_A_ synapses this depression of GABA_B_R-GIRK signaling persists for days after the injection (Padgett et al., 2012).

### Behavioural correlates of drug-evoked potentiation in VTA DA cells

As mentioned earlier the effect of non-contingent drug administration on synaptic plasticity in VTA DA neurons is transiently expressed, lasting at least 5 but less than 10 days and has been shown to positively correlate with the initial development of behavioral sensitization but not with its expression (Ungless et al., 2001; Saal et al., 2003; Borgland et al., 2004). In support of the hypothesis that drug-evoked potentiation of VTA synapses represents induction of behavioral sensitization, intra-VTA administration of glutamate antagonists reduce, and virally-mediated GluR1 up-regulation enhances the locomotor sensitizing properties of drugs (Carlezon et al., 1997; Carlezon and Nestler, 2002). Strong evidence of NR2A- and B-containing NMDA receptor involvement is provided by the observation that pharmacological inhibition of either prevents both the development of sensitization and associated cocaine-induced increases in AMPA/NMDA ratios (Schumann et al., 2009). However, mice with targeted deletion of NR1 or GluR1 (selective to midbrain DA neurons) or global GluR1 deletion exhibit intact behavioral sensitization and yet show impaired AMPA receptor currents after cocaine treatment (Dong et al., 2004; Engblom et al., 2008). An added twist is provided by the observation that CPP and conditioned locomotor behavior is absent in GluR1 knockout mice (Dong et al., 2004) and extinction of cocaine CPP is absent in mice with GluR1 deletion targeted to midbrain DA neurons (Engblom et al., 2008), whereas in NR1 knockout mice reinstatement of cocaine CPP and expression of behavioral sensitization is attenuated (Engblom et al., 2008; Zweifel et al., 2008). Thus, even with the caveat of potential developmental compensation in mutant mice and/or possible incomplete deletion, it is possible that that neural processes governing drug-evoked potentiation of DA neurons and behavioral sensitization are dissociated. Rather it may be that potentiation of VTA synapses may contribute to the attribution of incentive salience to drug-associated cues.

Measuring synaptic changes following non-contingent drug administration is limited with respect to informing on the actual disease state of addiction. More relevant to the human condition are studies where changes in synaptic plasticity are measured following contingent drug administration e.g., operant self-administration. In this regard, synaptic strengthening of VTA DA cells induced by self-administration of cocaine is uniquely persistent, lasting 3 months into abstinence and shown to be resistant to extinction training (Chen et al., 2008). Thus, though initially proposed to be a transient event, it appears that drug-evoked plasticity in the VTA has the capacity to be long-lasting, demonstrating that the method of administration (contingent versus non-contingent) is a critical determinant of its longevity. This is supported by the observation that yoked controls in this study did not show a similar increase in AMPA/NMDA ratio; suggesting it is the learning of the cue-reward or action-outcome association which is driving plasticity. In contrast, self-administration of food or sucrose under similar parameters produce increases in AMPA/NMDA ratios that persist for 7 but not 21 days into abstinence, demonstrably transient compared to that induced by cocaine (Chen et al., 2008). The lack of persistence of food-induced plasticity demonstrates that the change in synaptic strength induced by cocaine is not merely a neural representation of the instrumental or cue-reward learning processes involved in the operant self-administration paradigm *per se*, rather a drug-specific effect which potentially represents a pathological strengthening of drug-cue associations. As mentioned previously, cues predicting reward have also been found to cause an increase in AMPA/NMDA ratios in the VTA, though not as persistent, supporting a role for this modification of excitatory synaptic function in reward learning (Stuber et al., 2008).

Interestingly, the magnitude of the increase in the AMPA/NMDA ratio is similar regardless of the number of injections (single vs. multiple), administration protocol (contingent vs. non-contingent), and length of access (limited access vs. extended access) (Borgland et al., 2004; Chen et al., 2008; Mameli et al., 2009). This indicates that the increase in AMPA/NMDA ratio observed in VTA DA cells is potentially a permissive event, perhaps signaling “salience” as opposed to representing an initiation of underlying neuropathology which would presumably increase with continued exposure.

### Drug-evoked plasticity at excitatory synapses in the NAc

Unlike the VTA a single cocaine injection does not cause increases in synaptic strength in the NAc when measured 24 h later (Thomas et al., 2001; Kourrich et al., 2007). This observation and the bidirectional timescale which follows with repeated administration and withdrawal demonstrates that drug-induced plasticity in the NAc is markedly different from that observed in the VTA. Indeed, when repeated injections of cocaine are administered (so as to induce behavioral sensitization), a decrease in the AMPA/NMDA ratio is observed at NAc shell synapses when measured 24 h after the last administration (Kourrich et al., 2007). This synaptic depression from repeated cocaine appears to be linked to plasticity in the VTA; upon selective disruption of mGluR1 function in the VTA only a single injection of cocaine is then required to cause this same depression of NAc synapses (Mameli et al., 2009). The authors of this study postulate that enhanced excitation of VTA projections may facilitate the coincident release of DA and glutamate in the NAc through an enhanced release of DA. This may then shift the threshold for the induction of local plasticity in the NAc by affecting circuit excitability or by integrating intracellular signaling processes (Mameli et al., 2009).

The functional significance of the depression of NAc synapses during acute withdrawal is unclear at this stage. One possible explanation may be that depression of NAc medium spiny neurons (MSNs) reduces their response to natural rewarding stimuli, hence contributing to the anhedonia experienced during acute withdrawal. It could also be that the decrease observed in the AMPA/NMDA ratio may be result of membrane insertion of NR2B-containing NMDA receptors (thus increasing the denominator of the ratio) as new silent synapses are found to occur in the NAc shell upon cocaine exposure (Huang et al., 2009). Silent glutamatergic synapses, which express functional NMDA receptor-mediated currents in the absence of AMPA receptor-mediated currents, are thought to possess an increased capacity to undergo strengthening of synaptic transmission (Isaac et al., 1995). Once generated, these silent synapses may facilitate recruitment of AMPA receptors thereby enhancing excitatory synaptic transmission. This provides a possible mechanism to explain increases in the surface level of AMPA receptors and subsequent AMPAR/NMDAR ratio observed in the NAc during protracted withdrawal (Boudreau and Wolf, 2005; Boudreau et al., 2007; Kourrich et al., 2007; Conrad et al., 2008). NR2B-containing NMDA receptors in the NAc could also been involved in the formation of drug-context associations as siRNA knockdown of this subunit prevents morphine CPP in mice but not behavioral sensitization (Kao et al., 2011).

Unlike cocaine, a repeated regimen of intermittent ethanol exposure results in a potentiation of synapses in response to a previously LTD-inducing stimulation protocol when measured 24 h after the last exposure (Jeanes et al., 2011). This NMDA-dependent potentiation is transient as after a further 48 h of withdrawal it has dissipated and neither LTP nor LTD can be induced (Jeanes et al., 2011). The authors interpret such robust changes in NAc plasticity as an indicator of the potential importance of this process in ethanol-induced neuroadaptations. Moreover, unlike psychostimulants, ethanol can act at NMDA receptors so therefore has the capacity to directly influence glutamatergic signaling.

### Synaptic potentiation observed in the NAc after a period of withdrawal

In contrast to the depression observed during acute withdrawal, potentiation of NAc shell synapses is observed after 10–14 days of withdrawal from repeated cocaine or morphine administration (Kourrich et al., 2007; Wu et al., 2012). Moreover, after 7 days withdrawal from a single administration of cocaine, an increase in the amplitude of mEPSCs as well as a loss of LTP induced by high frequency stimulation (HFS) is found in both core and shell NAc neurons expressing the dopamine D_1_ receptor (Pascoli et al., 2012). This change in the ability to induce synaptic plasticity is referred to as metaplasticity. Cocaine-induced metaplasticity is also observed following withdrawal from cocaine self-administration. Thus, rats that have self-administered cocaine followed by 3 weeks of either extinction or abstinence display a marked *in vivo* deficit in the ability to develop LTP in the NAc core after stimulation of the PFC. This observation was accompanied by a leftward shift in the input-output curve suggesting potentiation of fEPSP amplitude (Moussawi et al., 2009). Potentiation of NAc synapses is also observed in the form of increased AMPA-mediated currents following an extended period of abstinence after self-administration (Conrad et al., 2008). Collectively, these data suggest that synaptic potentiation in the NAc develops either as a function of duration of withdrawal, or as a function of time since the first administration of cocaine. A recent study supports the latter interpretation as similar increases in the frequency of mEPSCs was observed in D_1_ receptor-expressing MSNs in mice despite the absence or presence of a protracted withdrawal period following repeated cocaine administration (Dobi et al., 2011). Therefore, it seems the events leading to the changes in glutamatergic transmission in the NAc take some time to develop.

The contribution of specific AMPA receptor subunits to this change varies according to the stage of withdrawal and the method of administration; 10–21 days into withdrawal from both passive and self-administration GluR2-containing AMPA receptors appear to be responsible for changes in AMPA transmission (Boudreau and Wolf, 2005; Boudreau et al., 2007; Kourrich et al., 2007; Ferrario et al., 2010) whereas beyond 21 days GluR2-lacking AMPA receptors are added to synapses. The latter finding appears to be the case only when cocaine is self-administered (Conrad et al., 2008; McCutcheon et al., 2011), though see (Mameli et al., 2009). Given the increased conductance of GluR2-lacking AMPA receptors it may be that their insertion occurs in response to the depression of NAc synapses caused by cocaine self-administration, thereby resulting in increased MSN responsiveness to excitatory inputs that trigger cocaine-seeking in the future. Indeed, blocking GluR2-lacking AMPA receptors in the NAc prevents expression of incubated cue-induced cocaine seeking (Conrad et al., 2008), and cocaine-seeking induced by either AMPA or cocaine is also blocked by injections of antisense oligonucleotides of GluR1 mRNA into the NAc (Ping et al., 2008).

### Drug challenge after withdrawal reverts synaptic potentiation to depression

The increase in synaptic strength and surface expression of AMPA receptor subunits induced by cocaine in the NAc after withdrawal from non-contingent administration is subsequently reversed upon administration of further cocaine injections (re-challenge) (Thomas et al., 2001; Boudreau et al., 2007; Kourrich et al., 2007; Ferrario et al., 2010). Thus, synaptic depression is once again observed in the NAc shell when measured 24 h after this cocaine injection (Thomas et al., 2001), though see (Pascoli et al., 2012). Behaviorally this appears to correlate with the expression of sensitization, and in the case of amphetamine at least, has been shown to be clathrin-mediated and reliant on GluR2-dependent endocytosis of postsynaptic AMPA receptors (Brebner et al., 2005). The decrease in surface expression of AMPA receptors following cocaine challenge is transient as within 7 days surface expression recovers to levels comparable to unchallenged cocaine-pretreated rats (Ferrario et al., 2010). As such, it appears that history of cocaine exposure and withdrawal can readily change the direction of synaptic plasticity in the NAc.

A direct link was recently made between the potentiation of cortico-accumbal synapses on D_1_ receptor-positive cells following 7 days withdrawal and the expression of sensitization. As mentioned previously, after 7 days withdrawal from a single administration of cocaine, these synapses are found to be potentiated in both the core and shell (as measured by an increase in mEPSC amplitude) and LTP induced by HFS is reduced. The same was not found for synapses on D_2_ receptor-positive cells (Pascoli et al., 2012). When reversed optogenetically *in vivo* via a protocol known to induce LTD, cortico-accumbal synapses on D_1_-receptor positive cells displayed reduced mEPSCs and the expression of locomotor sensitization was prevented. Importantly, the ability of HFS to induce LTP was restored to these neurons (Pascoli et al., 2012), thus demonstrating a direct link between this particular synaptic adaptation at cortico-accumbal synapses and the expression of sensitization to cocaine.

### Persistent impairments in NAc core plasticity underlie the transition to addiction

As mentioned above, it appears that cocaine induces metaplastic changes in NAc MSNs. The term “metaplasticity” was originally coined by Abraham and Bear to describe the change in the ability of synapses to undergo future plasticity (Abraham and Bear, 1996). Thus, a loss of LTD is observed in both the NAc core and shell 24 h following the end of cocaine self-administration; however after 21 days abstinence this deficit is found exclusively in the core (Martin et al., 2006). The same deficit is not found in yoked animals nor animals that have self-administered food, demonstrating it to be specific to the voluntary self-administration of cocaine and not associated with instrumental learning nor the cocaine exposure *per se* (Martin et al., 2006), thus raising the possibility that drug-induced metaplasticity in the NAc core may underlie the transition from casual use to compulsive drug-seeking behavior. The impairment in NAc synapses induced by cocaine self-administration may manifest in drug addicts as an inability to inhibit their behavior and thus prevent compulsive drug-intake.

Subsequent *in vivo* electrophysiological experiments support this hypothesis. Self-administered cocaine followed by extinction training was shown to induce metaplasticity which impaired the ability of PFC stimulation to produce LTP or LTD in NAc core MSNs (Moussawi et al., 2009). Moreover, administration of N-acetylcysteine, a drug which normalizes glutamate levels and reduces craving in addicts (Amen et al., 2011), was found to reverse this cocaine-induced metaplasticity and restore the ability to induce LTP or LTD (Moussawi et al., 2009). These findings have been extended to an animal model of relapse, the reinstatement model (see Table 1). Treatment with N-acetylcysteine was shown to attenuate reinstatement of drug-seeking induced by either cue or prime, an effect that persisted 2 weeks beyond cessation of treatment. Importantly, this attenuation was linked to its ability to restore synaptic strength to cortico-accumbal synapses (Moussawi et al., 2011).

These data provide a possible causal relationship between cocaine-induced plasticity at cortico-accumbal synapses and susceptibility to relapse, consistent with a glutamate homeostasis theory of addiction. Thus, a failure of the PFC to control drug-seeking behaviors can be linked to an enduring imbalance between synaptic and non-synaptic glutamate (Kalivas, 2009). Chronic cocaine results in reduced basal levels of glutamate due to down-regulation of the cystine-glutamate exchanger. This removes tone from presynaptic mGlu2/3 receptors located at cortico-striatal synapses which normally function to limit glutamate release (Kalivas, 2009). N-acetylcysteine inhibits drug-seeking by activating the cystine-glutamate exchanger, thereby increasing extrasynaptic glutamate and stimulating presynaptic mGluR2/3 receptors to reduce the glutamate release associated with drug-seeking (Kalivas, 2009). Given the strong link between mGluR2/3 regulation of both synaptic glutamate release and drug-seeking, the capacity of mGluR2/3 antagonist to inhibit N-acetylcysteine restoration of LTP is consistent with the possibility that normalizing cortico-accumbal plasticity is ameliorative in terms of relapse (Moussawi et al., 2009).

Further evidence supporting a key role for adaptations at NAc glutamatergic synapses in drug-seeking behavior is provided by observations that up-regulation of GluR2-lacking AMPA receptors mediate the incubation of cocaine craving seen after extended abstinence from cocaine (Conrad et al., 2008), and disrupting trafficking of GluR2-containing AMPA receptors in either the NAc core or shell attenuates the capacity of cocaine to reinstate extinguished drug-seeking behavior (Famous et al., 2008). Enhanced AMPA receptor-mediated transmission appears to be particularly relevant to drug-seeking. Thus, intra-NAc core administration of an AMPA receptor agonist promotes while an antagonist inhibits cocaine-seeking (Cornish and Kalivas, 2000) and similar results are found for both heroin (Lalumiere and Kalivas, 2008) and alcohol (Backstrom and Hyytia, 2004). Indeed, increased AMPA-mediated transmission is consistent with a critical role for prefrontal glutamate release NAc core in mediating reinstatement of drug-seeking behavior (McFarland et al., 2003; Kalivas et al., 2005).

Given this established role for increased AMPA-mediated glutamate in drug-seeking behavior, is potentially not surprising that primed reinstatement of heroin-seeking in rats was recently shown to require LTP-like increases in synaptic strength at cortico-accumbal synapses (Shen et al., 2011). This increase in synaptic strength was accompanied by changes in spine remodeling and required up-regulation of the NR2B subunit of the NMDA receptor (Shen et al., 2011). Further studies examining synaptic potentiation as a result of drug-seeking in the absence of a drug prime will provide insight into the exact synaptic changes elicited by the drug-seeking behavior itself.

By examining synaptic changes in the context of models of chronic self-administration and drug-seeking behavior following extinction or abstinence, it is more likely that experimental outcomes will reflect the changes occurring in the brains of drug addicts as opposed to the being the result of drug exposure alone. Nevertheless, while it is apparent that drug self-administration induces long-lasting changes in synaptic transmission, it is unknown whether these are non-specific adaptations that occur in all individuals exposed to drugs, or whether these changes occur specifically in individuals developing addiction. Pioneering work from the Piazza laboratory addressed this question by comparing synaptic transmission in the NAc of rats that had been classified as either “addict” or “non-addict” using DSM-IV criteria (Kasanetz et al., 2010). Cocaine self-administering rats were classified as “addicts” if they exhibited difficulty in limiting cocaine intake, increased motivation to seek the cocaine and continued use despite adverse consequences. It was found that after 17 days of cocaine self-administration, both “addict” and “non-addict” rats exhibited suppression of NMDA receptor-dependent LTD in the NAc. After 50 days of cocaine self-administration, NMDA receptor-dependent LTD was restored in “non-addict” rats, but these impairments persisted in the “addict” rats, despite no difference in the amount of cocaine these two groups were exposed to Kasanetz et al. (2010). These experiments provide compelling evidence that the transition to addiction may be associated with a form of “anaplasticity,” or an inability to counteract drug-induced impairments in synaptic plasticity.

It is apparent from the evidence reviewed above that exposure to drugs of abuse can induce long-lasting changes in synaptic strength in brain regions and circuits associated with drug reward (Hyman et al., 2006; Kauer and Malenka, 2007; Kalivas and O'Brien, 2008; Luscher and Malenka, 2011). In addition to the VTA and NAc, synaptic adaptations upon exposure to drugs have also been characterized in other components of the mesolimbic system including the PFC, bed nucleus of the stria terminalis and central amygdala (Dumont et al., 2005; Fu et al., 2007; Van Den Oever et al., 2008). However, given the above findings it appears that specific deficits in cortico-accumbal synapses of MSNs are the most relevant to addiction in humans.

## Transcriptional mechanisms of drug-induced plasticity

While it is clear that drugs of abuse are able to modify synaptic transmission in the mesocorticolimbic system, for stable alterations in neuronal functioning to be achieved, *de novo* protein synthesis is required (Kandel, 2001). Indeed, repeated drug exposure results in region-specific alterations in gene expression and it has been postulated that these changes may underlie some of the enduring behavioral abnormalities that characterize addiction (McClung and Nestler, 2003; Chao and Nestler, 2004). There are a number of mechanisms by which drugs of abuse are able to regulate gene expression, including activation and suppression of transcription factors, epigenetic mechanisms and induction of non-coding RNAs.

### Transcription factors

Transcription factors are proteins that bind to specific DNA sequences to regulate gene transcription by interacting with the RNA polymerase II complex (Mitchell and Tjian, 1989). Transcription factors can be induced or repressed in response to environmental stimuli, resulting in changes in gene expression and ultimately neuronal function. A number of transcription factors have been identified for their potential role in addiction because their expression and activation is regulated in the mesocorticolimbic pathway upon exposure to drugs of abuse. ΔFosB is one such transcription factor that has received particular attention due to its unusual stability. ΔFosB is a truncated splice variant of the FosB gene, and it shares homology with other Fos family members including c-Fos, FosB, Fra1, and Fra2 which all heterodimerise with Jun family proteins (c-Jun, JunB, or JunD) to form activator protein-1 (AP-1) transcription factors (Morgan and Curran, 1995). These other Fos family members are induced rapidly in the striatum in response to acute administration of psychostimulants, however due to their instability this expression is transient and returns to basal levels within hours (Graybiel et al., 1990; Young et al., 1991; Hope et al., 1992). Conversely, ΔFosB accumulates in the striatum following chronic drug administration, and its expression persists for several weeks after the last drug exposure (Hope et al., 1994; Nye et al., 1995; Nye and Nestler, 1996; Pich et al., 1997; Muller and Unterwald, 2005; McDaid et al., 2006). Data from behavioral experiments support a role for ΔFosB in some of the lasting effects imparted by drugs of abuse. Over-expression of ΔFosB in the striatum results in increased locomotor responses to both acute and chronic cocaine, and increases the reinforcing properties of both cocaine and morphine (Kelz et al., 1999; Colby et al., 2003; Zachariou et al., 2006), whereas inhibition of ΔFosB produces the opposite behavioral effects (Peakman et al., 2003). Due to its ability to increase the incentive motivational properties of drugs of abuse, this transcription factor has been proposed to represent a “molecular switch” that facilitates the transition to addiction (Nestler, 2008).

cAMP response element-binding protein (CREB) is another transcription factor that has been the focus of a considerable amount of research due to its proposed role in drug-induced plasticity (McPherson and Lawrence, 2007). CREB is expressed ubiquitously in the brain, and can be activated by a multitude of intracellular signaling pathways that culminate in its phosphorylation at serine 133 (Mayr and Montminy, 2001). Phosphorylated CREB (pCREB) stimulates the recruitment of CREB-binding protein (CBP) which facilitates the transcription of various down-stream genes (Arias et al., 1994). pCREB is rapidly induced in the striatum upon exposure to psychostimulants (Konradi et al., 1994; Kano et al., 1995; Walters and Blendy, 2001; Choe et al., 2002) and this is hypothesized to represent a homeostatic mechanism that counteracts behavioral responses to drugs of abuse (McClung and Nestler, 2003; Dong et al., 2006). Consistent with this, overexpression of CREB in the NAc shell reduces the rewarding properties of cocaine in a conditioned place preference (CPP) paradigm, whereas the opposite is observed upon inhibition of CREB in this region (Carlezon et al., 1998; Pliakas et al., 2001). Similarly, genetic knockdown or inhibition of CREB in the dorsal striatum confers increased sensitivity to the locomotor activating properties of psychostimulants, adding further support to this hypothesis (Fasano et al., 2009; Madsen et al., 2012).

While data from CPP experiments support the idea of CREB acting as a negative modulator of drug reward, at least with respect to cocaine, this may be an oversimplification. A number of studies using various techniques to alter CREB function in the NAc shell have revealed that inhibition of CREB reduces cocaine reinforcement in a self-administration paradigm (Choi et al., 2006; Green et al., 2010; Larson et al., 2011), whereas cocaine reinforcement is enhanced by CREB overexpression in this region (Larson et al., 2011). These divergent findings are probably due to fundamental differences between instrumental and Pavlovian conditioning procedures as well as voluntary *vs*. involuntary drug administration. CPP involves associative learning processes, and is thought to be an indirect measure of the hedonic properties of a drug rather than drug reinforcement *per se* (Bardo and Bevins, 2000). Voluntary drug self-administration can be influenced by a number of emotional factors, and the ability of CREB activity in the NAc to reduce responses to anxiogenic stimuli (Barrot et al., 2002) and attenuate depressive behavior (Pliakas et al., 2001) could influence the propensity to self-administer drug. Interestingly, deletion of CREB from the PFC results in decreased motivation to self-administer cocaine (McPherson et al., 2010), demonstrating that the effect of CREB manipulation upon behavior also varies for different brain regions. This is perhaps not surprising given that the CREB transcriptome differs markedly according to the cell type (Cha-Molstad et al., 2004) and it would therefore be important to identify the changes in gene expression occurring down-stream of CREB that contribute to these phenotypes. Complicating things further is the observation that CREB in the NAc shell is essential for nicotine CPP (Brunzell et al., 2009), suggesting that the mechanisms underlying conditioned nicotine reward differ from those underlying cocaine and morphine, which are both enhanced by CREB inhibition in the NAc shell (Carlezon et al., 1998; Pliakas et al., 2001; Barrot et al., 2002).

### Epigenetic mechanisms

Epigenetics has a number of definitions, but in neuroscience it is commonly defined as changes in gene expression that occur through modulation of chromatin which are not brought about by changes in the underlying DNA sequence (McQuown and Wood, 2010). Chromatin describes the state of DNA when it is packaged within the cell. The basic repeating unit of chromatin is the nucleosome, which consists of 147 base pairs of DNA wrapped around an octamer composed of pairs of the four core histones (H2A, H2B, H3, and H4) (Luger et al., 1997). The amino terminal tails of these core histones can undergo a number of post-translational modifications including acetylation, methylation, phosphorylation, ubiquitination, and sumoylation (Berger, 2007). The addition and removal of these functional groups from histone tails is carried out by a large number of histone modifying enzymes, including acetyltransferases, deacetylases, methyltransferases, demethylases, and kinases (Kouzarides, 2007). These histone modifications serve to signal the recruitment of transcription factors and other proteins involved in transcriptional regulation, and alter chromatin conformation to make DNA more or less accessible to the transcriptional machinery (Strahl and Allis, 2000; Kouzarides, 2007; Taverna et al., 2007). Epigenetic mechanisms therefore represent an important means by which environmental stimuli can regulate gene expression and ultimately behavior.

Recently, chromatin modification has been recognized as an important mechanism underlying drug-induced changes in plasticity and behavior (Renthal and Nestler, 2008; Bredy et al., 2010; McQuown and Wood, 2010; Maze and Nestler, 2011; Robison and Nestler, 2011). The first evidence for this came from experiments by Kumar and colleagues who used chromatin immunoprecipitation (ChIP) assays to demonstrate that cocaine induces histone modifications at specific gene promoters in the striatum (Kumar et al., 2005). Specifically, acute administration of cocaine resulted in H4 hyperacetylation of the *cFos* promoter, whereas chronic administration resulted in H3 hyperacetylation of the *BDNF* and *Cdk5* promoters. Histone acetylation involves the enzymatic transfer of an acetyl group to a histone's basic N-terminal tail, which neutralises the electrostatic interaction between the histone and the negatively charged DNA, making it more accessible to the transcriptional apparatus (Loidl, 1994). This is consistent with the ability of cocaine to increase the expression of Fos family transcription factors acutely (Graybiel et al., 1990; Young et al., 1991), whereas BDNF and Cdk5 are induced only upon chronic exposure (Bibb et al., 2001; Grimm et al., 2003).

A histone hyperacetylated state can also be achieved experimentally by administration of histone deacetylase (HDAC) inhibitors, and these drugs have been used to examine the effects of global increases in histone acetylation upon behavioral responses to drugs of abuse. Systemic administration of HDAC inhibitors synergistically increases the hyperacetylation observed in response to cocaine within the striatum (Kumar et al., 2005), and this potentiates cocaine-induced locomotion and cocaine reward (Kumar et al., 2005; Sun et al., 2008; Sanchis-Segura et al., 2009). HDAC inhibition can also increase locomotor sensitization to ethanol and morphine, and facilitate morphine CPP (Sanchis-Segura et al., 2009), Nevertheless, HDAC inhibitors have also been found to prevent the development of sensitization to a single morphine exposure (Jing et al., 2011), and reduce the motivation to self-administer cocaine (Romieu et al., 2008). These contrasting findings may reflect differences in administration protocols, and importantly they demonstrate that HDAC inhibitors do not indiscriminately potentiate behavioral responses to drugs in all conditions.

Due to their permissive effect upon gene transcription, HDAC inhibitors may also act to facilitate certain types of learning (Bredy et al., 2007; Lattal et al., 2007). It has recently been demonstrated that administration of a HDAC inhibitor following re-exposure to a previously cocaine-paired environment can facilitate extinction of cocaine-induced CPP, and this is probably related to increased histone H3 acetylation in the NAc (Malvaez et al., 2010). Infusion of the HDAC inhibitor suberoylanilide hydroxamic acid (SAHA) directly into the NAc during the conditioning phase of CPP increases conditioned cocaine reward (Renthal et al., 2007), indicating that HDAC inhibition in this region can facilitate both reward-related learning and extinction learning, depending upon the context in which the drug is administered. Further experiments have revealed a role for HDAC5, and endogenous HDAC expressed highly in the NAc in modulation of cocaine reward. Cocaine administration increases HDAC5 function by regulating its dephosphorylation and subsequent nuclear import, and dephosphorylation of HDAC5 in the NAc impairs the development of a cocaine CPP (Taniguchi et al., 2012). Similarly, over-expression of HDAC5 in the NAc during the conditioning phase of CPP attenuates cocaine reward, and this effect is reversed upon expression of a mutant form of HDAC5 in the NAc (Renthal et al., 2007). It is possible that HDAC5 is exerting these effects by inhibiting drug-induced gene transcription that normally increases the rewarding properties of cocaine.

Genome-wide analysis of chromatin modifications that occur in the NAc as a result of cocaine exposure has revealed a multitude of chromatin modifications at the promoter regions of genes down-stream of both CREB and ΔFosB (Renthal et al., 2009). This analysis also revealed up-regulation of two sirtuins, SIRT1 and SIRT2, which are proteins that possess HDAC activity and can also deacetylate other cellular proteins (Denu, 2005). Induction of SIRT1 and SIRT2 is associated with increased H3 acetylation and increased binding of ΔFosB at their gene promoters, suggesting that they are down-stream targets of ΔFosB (Renthal et al., 2009). The up-regulation of SIRT1 and SIRT2 is thought to have behavioral relevance; sirtuins decrease the excitability of NAc MSNs *in vitro*, and pharmacological inhibition of sirtuins decreases cocaine reward, whereas their activation increases rewarding responses to cocaine (Renthal et al., 2009).

In addition to the functional role for HDACs, genetic studies have also revealed a role for histone acetyltransferases (HATs) in mediating some of the behavioral responses to drugs of abuse. Arguably the most important mechanism by which CBP is able to enhance gene transcription is via its intrinsic HAT activity (Bannister and Kouzarides, 1996), and recent findings implicate the HAT activity of CBP in some of the epigenetic changes that result from drug exposure. In response to acute cocaine, CBP is recruited to the *FosB* promoter where it acetylates histone H4 and increases expression of FosB (Levine et al., 2005). In mice haploinsufficient for CBP, less CBP is recruited to the promoter resulting in decreased histone acetylation and FosB expression. This also corresponds to less accumulation of ΔFosB in the striatum, and not surprisingly these mice exhibit decreased sensitization in response to a cocaine challenge (Levine et al., 2005). Recently, using the cre-lox recombination system Malvaez and colleagues investigated the role of CBP activity located specifically in the NAc upon cocaine-induced gene transcription and behavior (Malvaez et al., 2011). It was reported that targeted deletion of CBP in the NAc resulted in reduced histone acetylation and c-Fos expression, and impaired locomotor activation in response to both acute and chronic cocaine (Malvaez et al., 2011). Conditioned cocaine reward was also inhibited in these mice, providing the first evidence that CBP activity in the NAc is important for the formation of drug-associated memories (Malvaez et al., 2011).

Recently, experiments from the Kandel lab have revealed that epigenetic mechanisms may underlie nicotine's hypothesized ability to act as a “gateway drug”. Mice chronically pretreated with nicotine prior to cocaine exposure exhibited enhanced locomotor sensitization and cocaine reward compared to nicotine naive mice (Levine et al., 2011). Additionally, nicotine pretreatment resulted in enhanced cocaine-induced depression of LTP in excitatory synapses in the NAc core, an effect that was not seen with nicotine alone. Analysis of histone modifications induced by 7-day nicotine exposure revealed increased H3 and H4 acetylation at the *FosB* promoter in the striatum, an effect that was not as pronounced in response to 7-day cocaine administration. HDAC activity was reduced in the striatum of nicotine treated mice, but unchanged in mice treated with cocaine. Remarkably, infusion of a HDAC inhibitor directly into the NAc was able to mimic the effects of nicotine pretreatment in potentiating cocaine's effects. None of these changes were observed when mice were treated with cocaine prior to nicotine, confirming the temporal specificity of these effects. This elegant set of experiments has provided a possible epigenetic explanation as to why cigarette smoking almost always precedes cocaine use in the human population (Kandel, 1975; Kandel et al., 1992).

In addition to histone acetylation, histone methylation has also recently been recognized as a behaviorally relevant chromatin modification induced by drugs of abuse (Laplant et al., 2010; Maze et al., 2010, 2011). Histone methylation involves the enzymatic addition of one, two, or three methyl groups to lysine or arginine residues at the N-terminal of histone tails, and is associated with either transcriptional activation or repression, depending upon the nature of the modification (Rice and Allis, 2001). The first studies to examine histone methylation induced by cocaine led to the identification of two histone methyltransferases, G9a and G9a-like protein (GLP), that were persistently down-regulated in the NAc 24 h following both non-contingent cocaine exposure and cocaine self-administration (Renthal et al., 2009; Maze et al., 2010). This down-regulation was linked to similar decreases in histone H3 lysine 9 (H3K9) and 27 (H3K27) methylation. Subsequently, G9a overexpression in the NAc was demonstrated to reduce cocaine-induced expression of selected genes, decrease cocaine reward as measured by CPP, and inhibit the increases in dendritic spine density normally observed in response to repeated cocaine (Maze et al., 2010). The opposite occurred when G9a expression in the NAc was inhibited, resulting in increased dendritic spine density and enhanced cocaine reward. There is evidence that these cocaine-induced changes in G9a expression and subsequent decreases in H3K9 and H3K27 are regulated by ΔFosB (Maze et al., 2010). Collectively, these experiments identified an important role for histone methylation by G9a in some of the long term behavioral and biochemical consequences of repeated exposure to cocaine.

Recently, trimethylation of histone H3 lysine 9 (H3K9me3) which was previously thought to be a relatively stable heterochromatic mark, was shown to be dynamically regulated in the NAc by acute and chronic cocaine exposure (Maze et al., 2011). Repeated cocaine resulted in persistent decreases in repressive H3K9me3 binding which was particularly enriched in non-coding genomic regions (Maze et al., 2011). These initial findings suggest that repeated cocaine exposure may lead to the unsilencing of certain retrotransposable elements in NAc neurons, and it would be of great interest to ascertain the behavioral consequences of these novel epigenetic adaptations.

Given the enduring nature of addiction, recent research has also explored the role of DNA methylation, which is a more stable epigenetic adaptation compared to histone modification. DNA methylation involves the addition of methyl groups to cysteine bases in DNA, and it is generally associated with transcriptional repression (Stolzenberg et al., 2011). Analysis of brains of rats that received passive cocaine injections over 7 days, or that self-administered cocaine over 13 days revealed down-regulation of the DNA methyltransferase DNMT3a in the NAc 24 h after the last cocaine exposure (Laplant et al., 2010). Conversely, following more chronic cocaine exposure (both passive and self-administered for 3 weeks or more) and a 28 day withdrawal period, *dnmt3a* mRNA was found to be significantly enhanced in the NAc (Laplant et al., 2010). Inhibition of DNA methylation/DNMT3a specifically in the NAc was subsequently shown to enhance both CPP and locomotor sensitization to cocaine, whereas the opposite was observed following overexpression of DNMT3a in this region. Moreover, inhibition of DNMT3a in the NAc also prevented cocaine-induced increases in dendritic spine density (Laplant et al., 2010). The behavioral relevance of cocaine-induced alterations in NAc spine density is still not well understood. Manipulations that inhibit drug-induced spine induction have been shown to reduce the rewarding properties of cocaine (Russo et al., 2009; Maze et al., 2010); however, other studies have found that inhibition of spinogenesis potentiates cocaine reward (Pulipparacharuvil et al., 2008; Laplant et al., 2010). As cocaine appears to induce a highly complex regulation of various dendritic spines over the course of exposure and withdrawal (Shen et al., 2009), it has been suggested that these differences may depend upon the type of dendritic spines that are altered (Laplant et al., 2010).

From the experiments described herein, it is clear that drug-induced regulation of the transcriptional potential of cells represents a key mechanism influencing behavioral responses to drugs and reward-related learning. An important next step would be to identify which of these epigenetic changes are most relevant to the human disease state of addiction. Given that mere exposure to drugs is insufficient to produce “addiction” in both humans and animals, the incorporation of models that more closely measure behavioral hallmarks of addiction, such as compulsive drug use and relapse will be of significant value.

### MicroRNAs

MicroRNAs represent yet another important means by which drugs of abuse can regulate gene expression. MicroRNAs are small, non-coding RNA transcripts that act to inhibit gene translation at the post-transcriptional level by targeting the 3′-untranslated region (3′UTR) (Bartel, 2004). Recent work by Paul Kenny's group has lead to the identification of transcriptional regulation by microRNAs that occurs specifically in rats with extended access to cocaine self-administration (Hollander et al., 2010; Im et al., 2010). Extended access models precipitate escalating, compulsive patterns of drug-intake which is thought to be reminiscent of the uncontrolled drug use that characterizes human addiction (Ahmed and Koob, 1998; Deroche-Gamonet et al., 2004; Vanderschuren and Everitt, 2004). In rats with a history of extended access to cocaine, the microRNA miR-212 was up-regulated in the dorsal striatum (Hollander et al., 2010), a brain region that becomes progressively engaged with prolonged drug experience (Letchworth et al., 2001; Porrino et al., 2004). Virally-mediated over-expression of miR-212 in the dorsal striatum decreased the motivation to consume cocaine, but only under extended access conditions (Hollander et al., 2010). Inhibition of miR-212 signaling in this region produced the opposite effect, and facilitated compulsive cocaine self-administration. miR-212 is induced in response to CREB signaling (Vo et al., 2005), and exerts its effects by potentiating the activity of CREB (Hollander et al., 2010), revealing a novel feedforward mechanism whereby miR-212 is seemingly able to protect against the development of compulsive cocaine intake.

Expression of the transcription factor MeCP2 is also specifically increased in the dorsal striatum of rats following extended access to cocaine (Im et al., 2010). Disruption of MeCP2 activity in the dorsal striatum prevents the escalation of drug intake normally seen in extended access rats, and results in a progressive decline in responding for cocaine. Unlike CREB and ΔFosB, MeCP2 is a transcriptional repressor, exerting its effects by recruiting HDACs and other transcriptional repressors to silence target genes (Nan et al., 1998). MeCP2 acts to repress expression of miR-212 in the dorsal striatum in an activity dependent manner, and also controls the expression of brain-derived neurotrophic factor (BDNF), a protein with an established role in modulating cocaine-related behaviors (Horger et al., 1999; Graham et al., 2007). miR-212 can also feedback to repress expression of MeCP2, and these two transcriptional regulators are involved in a negative homeostatic balancing act (Im et al., 2010).

These studies highlight the complexity of transcriptional regulation that occurs as a result of drug self-administration, and suggest that voluntary drug intake is controlled by a fine balance of opposing molecular regulators that act to facilitate or inhibit compulsive drug use. It would be of great interest to ascertain whether transcriptional regulation by miR-212/MeCP2 is involved in the mechanism of “recovery” observed in non-addict rats (Kasanetz et al., 2010), and this may bring us closer to understanding factors that underlie both vulnerability and resilience to addiction (Ahmed, 2012).

## Conclusions

Research over the last decade has provided insight into the ability of drugs of abuse to modify synaptic transmission within mesocorticolimbic and corticostriatal circuitry, and we are now beginning to unravel the behavioral significance of some of these changes. More recently, the growing field of epigenetics has shed light upon some of the mechanisms by which drugs of abuse regulate the transcriptional potential of cells, to initiate lasting changes in gene expression. This research has opened up several potential therapeutic avenues. The discovery that N-acetylcysteine is able to restore synaptic deficits induced by self-administration of cocaine, and inhibits reinstatement of drug-seeking offers promise for “rehabilitated” addicts (Moussawi et al., 2011). HDAC inhibitors are gaining attention for their ability to enhance certain types of learning, and the recent discovery that sodium butyrate can facilitate extinction of a cocaine-induced CPP and attenuate reinstatement of drug-seeking is promising (Malvaez et al., 2010). An important next step would be to interrogate the ability of HDAC inhibitors to facilitate extinction of operant self-administration, which more accurately models voluntary drug consumption in humans. Finally, the identification of factors that regulate escalating drug use both on a synaptic level (e.g., persistent impairments in NMDAR-dependent LTD in the NAc) and on a molecular level (e.g., striatal signaling pathways involving miR-212 and MeCP2) are bringing us closer to understanding the mechanisms that underpin the transition to addiction (Hollander et al., 2010; Im et al., 2010; Kasanetz et al., 2010). These studies highlight the importance of examining neuroplastic changes that are brought about by voluntary drug self-administration rather than passive drug exposure. Moving forward it would be important for more research to incorporate these self-administration models that more closely mimic the behavioral pathology seen in human addicts.

### Conflict of interest statement

The authors declare that the research was conducted in the absence of any commercial or financial relationships that could be construed as a potential conflict of interest.
